# Segregation of chromosome arms in growing and non-growing *Escherichia coli* cells

**DOI:** 10.3389/fmicb.2015.00448

**Published:** 2015-05-12

**Authors:** Conrad L. Woldringh, Flemming G. Hansen, Norbert O. E. Vischer, Tove Atlung

**Affiliations:** ^1^Bacterial Cell Biology, Faculty of Science, Swammerdam Institute for Life Sciences, University of AmsterdamAmsterdam, Netherlands; ^2^Department of Systems Biology, Technical University of DenmarkLyngby, Denmark; ^3^Department of Science, Systems and Models, Roskilde UniversityRoskilde, Denmark

**Keywords:** *Escherichia coli*, nucleoid, DNA segregation, chromosome arms (replichores), ordering pattern, rifampicin-treatment, run-off DNA synthesis

## Abstract

In slow-growing *Escherichia coli* cells the chromosome is organized with its left (L) and right (R) arms lying separated in opposite halves of the nucleoid and with the origin (O) in-between, giving the pattern L-O-R. During replication one of the arms has to pass the other to obtain the same organization in the daughter cells: L-O-R L-O-R. To determine the movement of arms during segregation six strains were constructed carrying three colored loci: the left and right arms were labeled with red and cyan fluorescent-proteins, respectively, on loci symmetrically positioned at different distances from the central origin, which was labeled with green-fluorescent protein. In non-replicating cells with the predominant spot pattern L-O-R, initiation of replication first resulted in a L-O-O-R pattern, soon changing to O-L-R-O. After replication of the arms the predominant spot patterns were, L-O-R L-O-R, O-R-L R-O-L or O-L-R L-O-R indicating that one or both arms passed an origin and the other arm. To study the driving force for these movements cell growth was inhibited with rifampicin allowing run-off DNA synthesis. Similar spot patterns were obtained in growing and non-growing cells, indicating that the movement of arms is not a growth-sustained process, but may result from DNA synthesis itself. The distances between loci on different arms (LR-distances) and between duplicated loci (LL- or RR-distances) as a function of their distance from the origin, indicate that in slow-growing cells DNA is organized according to the so-called sausage model and not according to the doughnut model.

## Introduction

The chromosome of *Escherichia coli* can be readily visualized in living cells by phase contrast microscopy as a separate and dynamic structure (Mason and Powelson, 1956; Yamaichi and Niki, 2004). We now know that within this nucleoid structure the DNA is confined as a single branched, plectonemic supercoil formed and maintained by topoisomerases (Zechiedrich et al., 2000) and by nucleoid associated proteins (NAPs; Luijsterburg et al., 2006). The primary cause for the phase separation between nucleoid and cytoplasm is the physical phenomenon of excluded-volume interactions between DNA and soluble proteins (Odijk, 1998). Studies of the physical structure of isolated bacterial DNA (Cunha et al., 2001; Pelletier et al., 2012) have indicated that the DNA segments behave as entropic springs showing diffusive motion within a visco-elastic network (Cunha et al., 2005).

Against this physical background we must consider the process of bacterial segregation and the dynamics of replicated DNA strands collapsed or confined into the nucleoid of living cells. Do these strands become entangled or mixed as is to be expected for such polymer chains? It is now well accepted that DNA daughter strands segregate as they are replicated and that in slow growing *E. coli* cells the two chromosome arms move to different halves of the nucleoid with the origin in-between (Nielsen et al., 2006a; Wang et al., 2006). This organization suggests that the replicated daughter strands do not entangle or mix, but stay separated as Left and Right arms of the chromosome during the entire replication-segregation process (review Jun and Wright, 2010).

Can such an organization and movement be explained without the help of an underlying biological structure as suggested by several authors (Wiggins et al., 2010; Le Chat and Espéli, 2012; Yazdi et al., 2012), a hypothetical structure that, in its turn, has to become organized? This question has recently been considered by Youngren et al. (2014) for the even more complicated situation in fast growing *E. coli* cells undergoing multifork replication. These authors propose that in the wider cells replicating chromosomes are thermodynamically driven into ring polymers in which replicated strands segregate spontaneously by entropic demixing without the help of any additional, biological mechanism.

Nevertheless, it has been proposed that segregation proceeds in growing cells with the help of transcription/transertion processes (Woldringh, 2002). In addition, it was suggested that the demixing process may be sustained by regulatory interactions between transcription factors and target genes that help to self-organize the chromosome into topological domains that do not mix (Fritsche et al., 2011). According to Fisher et al. (2013), the non-intermingling of sister strands occurs in pulses of nucleoid elongation at defined times in the cycle of slowly grown cells while cell elongation continues monotonically. The periodical movements are proposed to depend on the accumulation and release of tethers between sister strands, processes that can be expected to depend on cell growth.

In view of these growth-sustained, active processes proposed to organize and segregate the replicating chromosome we analyzed the positions of fluorescently tagged loci in either growing *E. coli* cells or in rifampicin-treated cells that do not grow but only carry out run-off DNA synthesis. The measurements indicate that separation and migration of loci is very similar in growing and non-growing cells suggesting that the process of segregation continues in the absence of RNA synthesis without cellular or nucleoid elongation. The measurements are discussed in the light of two segregation models proposed in the literature, the so-called doughtnut and sausage model.

## Materials and methods

### Strains and growth conditions

**Figure 1:**
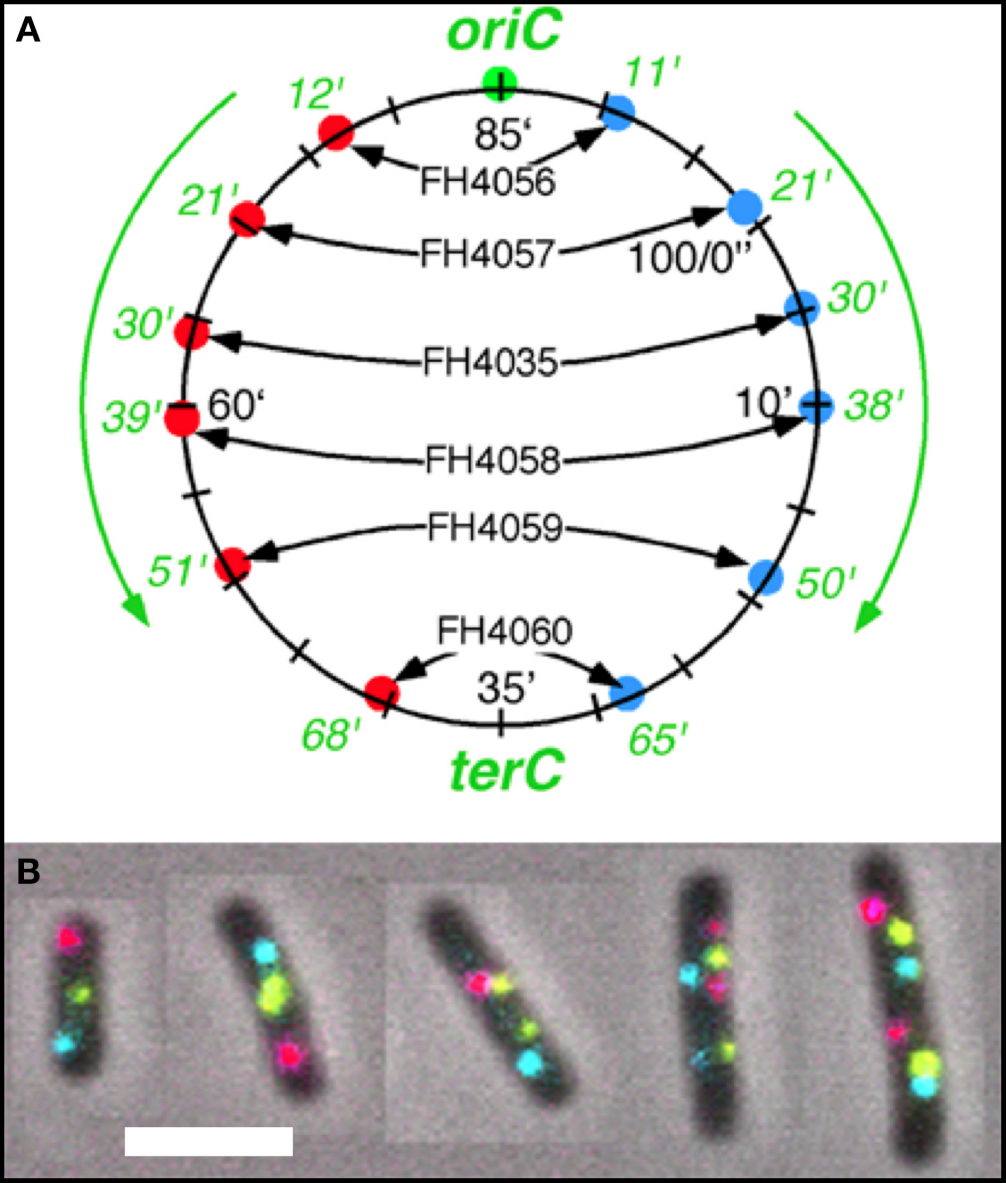
**Fluorescent loci and spots. (A)** Schematic representation of the chromosome of the 6 strains showing the positions of the 3 probes. Green fluorescent protein labels a locus ~1 kb to the right of the origin (O). The chromosome arms are labeled, respectively, with red (L, left arm) and cyan (R, right arm) fluorescent protein on opposite sites and at different distances from *oriC*. Green numbers indicate the theoretical time when the loci are replicated during progression of the replication forks, based on their distance in kb from *oriC* and on the replication velocity, assuming the replication time to be 75 min (Nielsen et al., 2006a). **(B)** Composite image of the 4 channels (phase contrast, red, green, and cyan filters) of cells of *E. coli* FH4035 showing the most abundant pattern of 3-spot cells (LOR) and 6-spot cells (LOR LOR), as well as some replicative intermediates. Magnification bar 2 μm.

Strains of *E. coli* MG1655 Δ*lacIZYA* (Nielsen et al., 2006b) carrying three different ParB/*parS* systems were constructed by recombineering. The strains with the three different *parS* sequences were transformed with plasmid pFH4034 which carry three different *parB* gene fusions to three different genes for fluorescent proteins with different colors (green (G), cyan (C), and red (R); see Supplementary Data Figure S1) to give the strains depicted in Figure 1A. See for strain construction Supplementary Material and Table S1.

The strains were grown in minimal glycerol medium supplemented with lysine (20 μg/ml) and ampicillin (100 μg/ml). Strain FH4035 has a lysine requirement while the strains FH404056-4060 are *lysA*^+^. We add 10 μg/ml uracil due to a possible mutation in the *rph* gene which might affect the expression of the *pyrE* gene. Cell growth was monitored with a spectrophotometer at 450 nm. Cells were grown undisturbed at 32°C (doubling time about 150 min) for about 16 h while the OD was kept below 0.5 by periodical dilutions.

Strain FH4035 was grown in a slightly different growth medium at 28°C, with a doubling time of ~180 min. For unknown reasons these cells were shorter than those of the other constructs while showing the same cell diameter (0.7 μm). The smaller cell length leading to smaller segregation distances is evident from the Tables, but in our view does not influence our interpretation of patterns and movement of the chromosome arms.

### Microscopy

Cell samples (1 ml) were concentrated about 20x by centrifugation, prepared on an agar slab or a poly-lysine coated microscope slide and photographed within 30 min after sampling.

For the constructs FH4056, 4057, 4058, 4059, and 4060 pictures were taken on a Zeiss Axioplan microscope equipped with a Kappa DX 2 camera using an ImageBase capture program. For construct FH4035 an Olympus BX microscope was used in combination with programs ImageJ and MicroManager. Both microscopes had 100 × 1.3 oil immersion lens (PH3) giving a magnification of 15 pixels per μm. Four pictures of each field were obtained using the respective capture programs. Both microscopes had filters for detecting red, cyan, and green fluorescence.

### Measurement of cells and spots

Image analysis was performed with Coli-Inspector, which is a set of scripts packed in a “project file” running under the ImageJ plugin ObjectJ. After spatial alignment of the 4 channels, cell length and diameter were automatically measured. The positions of the three kinds of spots were automatically measured using different thresholds. Cells in which one kind of spot was lacking were manually discarded. In some cells missing spots could be manually added after better adjusting brightness and contrast of the image. After checking all cells by eye, the spot positions along the long and short axis of the cell, their pattern, the frequency of each pattern and the (half-) distance between duplicated spots were calculated by Coli-Inspector (see: https://sils.fnwi.uva.nl/bcb/oblectj/examples/). By making use of the so-called Qualifier-possibility the distribution of, for instance, cells that show a specific spot pattern can be obtained (cf. **Table 3**).

## Results

### Analysis of three- and four-spot patterns in growing cells

The segregation of chromosome arms in slowly growing *E. coli* cells (doubling time at 28°C or 32°C Td = 180/150 min), was studied by measuring the cellular positions of three fluorescently labeled loci. The chromosomes contain one locus of green fluorescent-protein near *oriC* (O) and two loci on opposite positions on the chromosome arms at different distances from *oriC* in six constructs. Red fluorescent-protein tags the left arm (L) and cyan fluorescent-protein the right arm (R). See Figure 1A for a schematic representation of the chromosome in the different strains. Figure 1B shows composite images of the 4 channels for typical cells of *E. coli* FH4035.

In non-replicating cells with only 3 spots the most prominent pattern is LOR (46–78% of cells containing 3 spots; Table 1A, column I). This reflects the occurrence of unmixed chromosome arms in the two halves of the nucleoid as originally described by Nielsen et al. (2006b) and Wang et al. (2006). However, in many cells the origin is lying outside the two arms, giving the patterns OLR or ORL (column II in Table 1A). This pattern is more prominent in the constructs with loci close to the terminus (strains FH4059 and FH4060), where it increases to 50 per cent of the cells. It does not necessarily mean that the origin lies at the end of the nucleoid as depicted in the cells of Table 1A. In these constructs (FH4059 and FH4060), origin proximal loci may well have passed the origin. On average the origin in the 3 spot cells showing the LOR configuration is located in the middle of the cell with a standard deviation of 0.08–0.16 (Figure S2A) in agreement with a previous study (Nielsen et al., 2006a).

**Table 1A:** **Percentages of most abundant patterns in non-replicating cells with 3 spots (column I and II) and in replicating cells with 4 spots that have a replicated origin (O; column III–V)**.

**Strains**	**Total number cells with spots (mean cell length, μm)**	**Number cells with 3 spots (% of total population)**	**Non-replicating cells. % of 3-spot cells (mean cell length, μm)**		**nr. cells with 4 spots (% of total population)**	**Replicating cells with only the origin (O) duplicated. % of 4-spot cells (mean cell length, μm)**				**nr cells with 5 spots (% of total population)**
			**I LOR**	**II OLR/ORL**		**III-A LOOR**	**III-B OOLR/OORL**	**IV OLOR/OLOR**	**V OLRO**	
			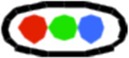	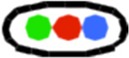		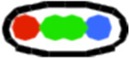	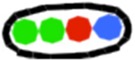	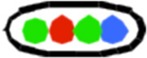	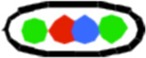	
4056^a^ 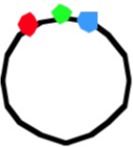	1376 (3.27)	527 (33)	62 (2.61)	38 (2.56)	63 (5)	35 (3.27)	6 (2.65)	40 (3.04)	19 (2.89)	80 (6)
4057^a^ 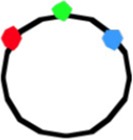	1099 (3.30)	487 (44)	74 (2.75)	26 (2.63)	138 (13)	32 (3.21)	9 (3.11)	42 (3.21)	15 (3.17)	56 (5)
4035^b^ 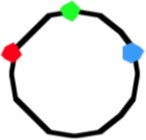	1003 (2.55)	384 (38)	77 (2.07)	23 (2.57)	129 (13)	36 (2.27)	1 (2.31)	36 (2.33)	26 (2.36)	48 (5)
4058^a^ 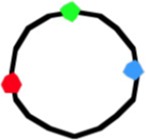	1166 (3.04)	617 (53)	78 (2.61)	22 (2.54)	191 (16)	44 (3.03)	8 (2.88)	35 (3.14)	13 (3.25)	33 (3)
4059^a^ 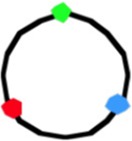	705 (3.09)	448 (64)	46 (2.75)	54 (2.80)	56 (8)	43 (3.16)	7 (2.75)	32 (3.48)	20 (3.38)	41 (6)
4060^a^ 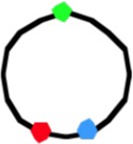	989 (2.98)	306 (31)	50 (2.49)	50 (2.37)	412 (42)	14 (2.92)	11 (2.83)	43 (2.88)	32 (3.17)	85 (9)

*The average cell length (μm) in each subpopulation is given within brackets*.

a *Cells grown at 32°C; doubling time Td = ~150 min*.

b *Cells grown at 28°C (Td = 180 min) showing smaller cell lengths (see Materials and Methods)*.

**Figure 2:**
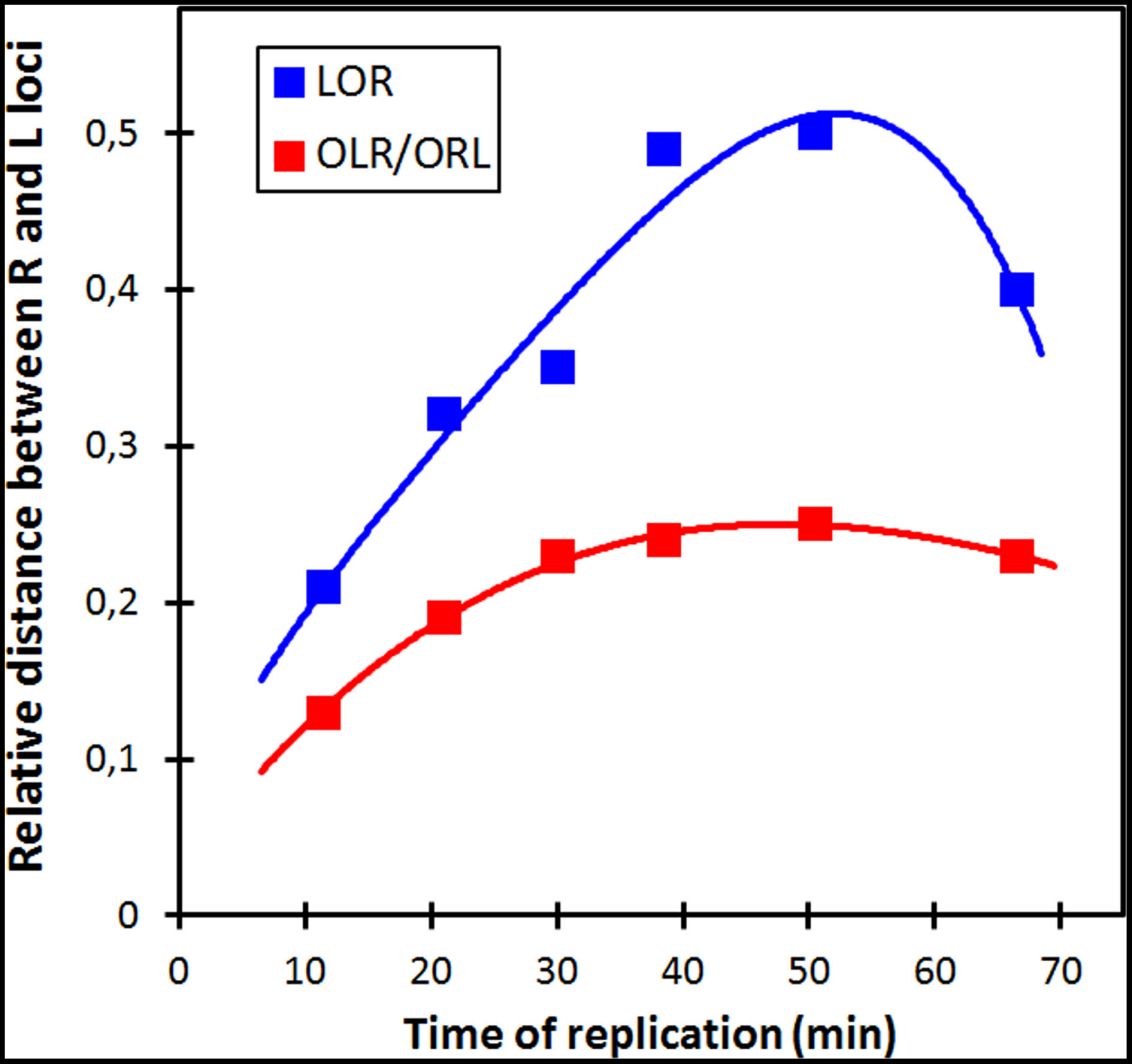
**Relative distances between L and R loci for the two different configurations of 3-spot cells**. See Table S2 for SD values. For each strain the distances between the L and R spots in the two different configuration types were measured and normalized to the average cell length. The distances are plotted as a function of the time of replication of the loci (see legend to Figure 1A).

We measured the distance between the L and R loci in the 3 spot cells (Figure 2, Table S2). The distances were small for the origin proximal loci increasing with increasing distance from the origin, except for the most terminus proximal pair; these two loci are closer together, but not as close as for the origin proximal one's and with larger cell to cell variation (Figure S2). The trends were the same for LOR cells and for OLR/ORL cells, but L-R distances were shorter for the latter cells in all six constructs. Also in 4-spot cells the L-R distances were found to increase for the 4 first constructs in all ordering patterns (see Table S3).

Initiation of DNA replication occurs after a significant increase in cell length (compare lengths indicated in columns I and II with those in columns IIIA and B in Table 1A) and results in cells with 4 spots. In general, the duplicated O-spots are found adjacent to each other, either in between the L- and R-arm spots (column III-A) or in rare cases on the outside (column III-B in Table 1A). The relatively low percentage of this latter pattern suggests that the origins soon move apart and pass either one or both of the other labeled loci giving the patterns as depicted in column IV and V in Table 1A. The relatively high percentage of cells in which the duplicated O-spots occur on the outside of spots on both chromosome arms in some of the constructs (column V in Table 1A) shows that some unreplicated loci stay relatively close to the mid of the long axis of the cell. It should be kept in mind that for the constructs with origin proximal loci 4 spot cells are a fairly homogeneous cohort, whereas the 4 spot cells of constructs with origin distal loci are a mixture of cells with different amounts of replicated DNA.

Analysis of the O-O distances (Table S3) as a function of LR locus position shows that soon after initiation the origins are lying close together (strains FH4056 and FH4057), and when later replicated loci are duplicated the origins have moved further apart (strains FH4035 and FH4058). Comparison of the L-R distances in the 3-spot cells (Figure 2) with the distances in the 4-spot cells (Table S3) suggests that upon initiation the un-replicated LR loci first move apart (LOOR pattern), but move inward again when the origin passes one (OLOR/OROL) or two (OLRO) arms, causing smaller distances. Table S3 further indicates that while the distance between origins (O-O) increases gradually in the origin-distal constructs, the L-R distances show, after an initial increase, a decrease in the terminal-proximal constructs (FH4059 and FH4060). This could be ascribed to the association of the terminus region with the divisome (Espéli et al., 2012).

Cells with a 5-spot pattern are obtained when a locus on only one of the chromosome arms has duplicated. The relatively low percentage of these cells (last column in Table 1A) indicates that the oppositely positioned loci on the two arms are replicated and segregated more or less synchronously, directly giving rise to the cells with 6 spots, discussed in the next section.

### Analysis of six-spot patterns in growing cells

Table 1B presents the cells which have replicated and segregated the three loci thus containing 6 spots. The spot patterns appeared to be very variable. With the 3 spots doubling to 6 spots, a range of 40–70 different patterns were obtained for the six constructs. Except for strain FH4060 the most predominant patterns are those shown in columns VI and VIII of Table 1B. The pattern in column VIII will upon division give rise to LOR cells shown in column I of Table 1A and the patterns shown in column VI will give rise to the 3-spot patterns shown in column I (LOR) and II (OLR/ORL) of Table 1A.

**Table 1B:** **Percentages of most abundant patterns of 6 spots in cells with replicating or replicated chromosomes of the different constructs (see Figure 1A)**.

**Strains**	**Number cells with 6 spots (mean cell length, μm)**	**% of total population**	**Percentage of 6-spot patterns**					
			**VI ORL ROL/OLR LOR**	**VII OLR LRO**	**VIII LOR LOR**	**IX LOR RLO/ROL LRO**	**X ROL LOR/LOR ROL**	**Other combi-nations (% of total population)**
			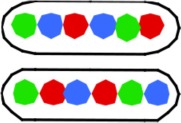	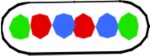	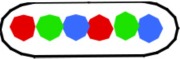	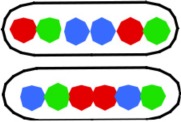	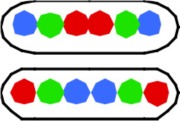	
4056^a^ 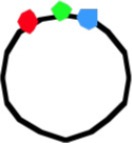	603 (3.80)	44	20 (3.85)	5 (3.64)	22 (3.92)	10 (3.67)	9 (3.96)	34 (2)
4057^a^ 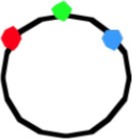	318 (4.04)	29	27 (4.11)	4 (3.92)	28 (4.19)	8 (3.99)	8 (4.04)	25 (2)
4035^b^ 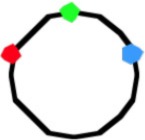	412 (3.02)	41	23 (2.89)	7 (2.79)	22 (3.23)	17 (3.02)	15 (3.10)	16 (2)
4058^a^ 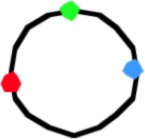	243 (3.89)	21	28 (3.82)	7 (3.60)	24 (4.07)	14 (3.88)	10 (4.31)	17 (2)
4059^a^ 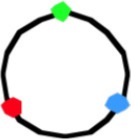	120 (3.97)	17	15 (4.05)	5 (3.75)	15 (3.82)	12 (4.16)	7 (4.14)	46 (7)
4060^a^ 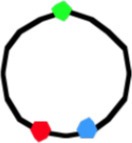	132 (3.85)	13	32 (4.05)	21 (3.66)	11 (4.24)	12 (4.01)	0	24 (2)

*The average cell length (μm) in each subpopulation is given within brackets*.

a *Cells grown at 32°C; doubling time Td = ~150 min*.

b *Cells grown at 28°C (Td = 180 min) showing smaller cell lengths (see Materials and Methods*.

Cells which still have the origins lying outside (ORL LRO) occur in relatively low percentages (column VII in Table 1B) and are in all cases the smallest of the 6 spot cells. This suggests that origins which are located at the quarter position relatively early in the cell cycle soon are passed by one or both of the newly replicated loci. Only in construct FH4060, where the loci lie close to the terminus, many of the duplicated L- and R- spots did not pass the origins probably reflecting that these loci, which have the terminus located in between them, remain close for some time after replication. This may reflect a slow movement of loci in the terminal region of the nucleoid up to the division of the cell.

Patterns where the loci on either the Left or Right arm remain adjacent are less frequent (columns IX and X in Table 1B). Patterns where loci on both the Left and Right arm remain adjacent (e.g. OLL RRO) are rarely observed (<1% of the cells with 6 spots), indicating a fast separation of duplicated spots along the length axis of the cell, thereby passing loci on the other chromosome arm. However, patterns with adjacent LL or RR spots do occur with an average of 4% in subpopulations of long cells (>3.5 μm). This could indicate that such cells may have experienced an unusual difficulty in replication (and thus segregation) or an extended cohesion time (see Wang et al., 2008), that postponed cell division.

While a minority of the cells show either ajacent loci on the Left or Right arm (columns IX and X in Table 1B), cells with all three duplicated loci remaining next to each other (LLOORR) have not been observed. We conclude that both the origin and the loci at different positions on the chromosome arms separate soon after their replication. The observed patterns are well in agreement with movements of chromosome arms as previously described by Nielsen et al. (2006a,b) and Wang et al. (2006).

### Segregation patterns during run-off DNA replication in non-growing cells

When cell growth is stopped by inhibiting protein synthesis (Maaløe and Hanawalt, 1961) or RNA synthesis (Lark, 1972), ongoing rounds of DNA replication terminate as is seen by both flow cytometry (Michelsen et al., 2003; Nielsen et al., 2006a), and image cytometry (Huls et al., 1999) of similar samples. To investigate whether segregation of chromosome arms still takes place under such conditions, spot patterns were determined in cells of *E. coli* strain FH4035 treated with 300 μg/ml rifampicin for 210 min at 28°C (cf. Skarstad et al., 1986). If replicated spots would not segregate in rifampicin treated cells we would expect to observe a high percentage of cells with adjacent spots (LLOORR).

**Figure 3:**
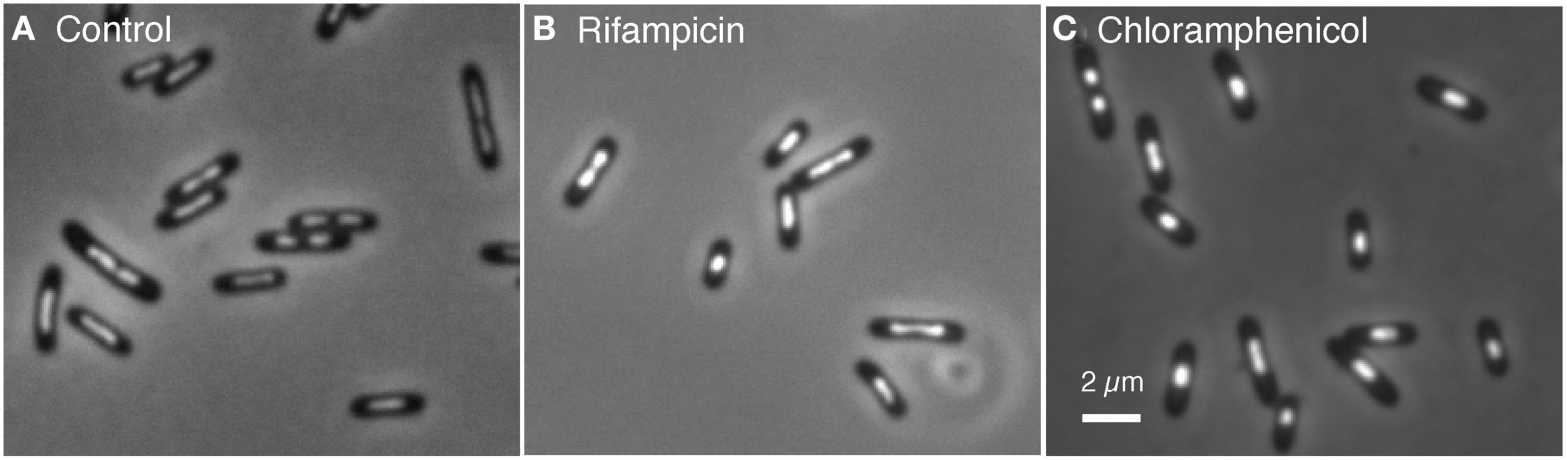
**Cells of *E. coli* FH4035, grown in glycerol minimal medium at 28°C, were treated during 100 min with the indicated inhibitors, fixed with 0.1% OsO4 and stained with 10 μg/ml DAPI. (A)** In the control cells divided and well separated nucleoids occur in 15% of the cells. **(B)** In rifampicin-treated cells only 9% of the cells show divided nucleoids. **(C)** In chloramphenicol-treated cells the nucleoids (only 3% divided) have a more spherical shape.

For comparison of nucleoid sizes and shapes, cells were also treated with chloramphenicol under the same conditions. Figure 3 shows the various cell samples fixed with osmium tetroxide and stained with DAPI for visualizing and measuring nucleoids. In contrast to the control cells showing a skewed distribution of nucleoid lengths (average 1.21 μm), the rifampicin-treated cells showed a bimodal distribution with peaks at 0.77 and 1.5 μm (results not shown). It seems probable that these peaks represent 1 and 2 chromosome equivalents.

We performed three independent growth experiments in which cells of strain FH4035 were treated with 300 μg/ml rifampicin for 210 min. These populations were analyzed for spot patterns and the combined results are shown in Table 2, together with the data of strain FH4035 given in Tables 1A,B, as a reference.

**Table 2:** **Percentages of most abundant patterns in cells of strain FH4035, during growth (cf. Tables 1A,B) and after inhibition with rifampicin (300 μg/ml for 210 mi**.

**Cells** 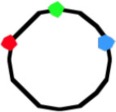	**Total nr. cells with spots**	**% cells with 3 spots**	**% cells with 4 spots**	**% cells with 5 spots**	**% cells with 6 spots**	**Percentage of patterns in cells with 6 spots**					
						**VI ROL RLO/LOR LRO**	**VII ORL RLO**	**VIII LOR LOR**	**IX ORL LOR/OLR ROL**	**X ROL LOR/LOR ROL**	**Other combinations**
						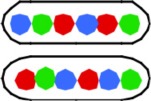	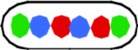	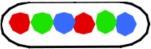	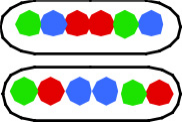	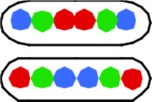	
Control^a^	1003 (2.55)	38	13	5	41	23 (2.89)	7 (2.79)	22 (3.23)	17 (3.02)	15 (3.10)	16
Rifampicin^b^	2065 (2.37)	46	1	1	49	13 (2.69)	2 (2.34)	25 (2.86)	12 (2.72)	20 (2.82)	28

*The average cell length (μm) for each subpopulation s given within brackets*.

a *Strain FH4035; compare with data in Tables 1A,B*.

b *Cells of strain FH4035 treated with rifampicin; data from three independent growth experiments have been averaged*.

Rifampicin treatment caused a decrease of average cell length from 2.55 to 2.37 μm. This can be attributed to residual divisions, also indicated by the decrease in the percentage of cells with divided nucleoids (from 15 to 9%) and of constricted cells from 14% in the control to 3% in the rifampicin-treated samples (results not shown). Also because of residual division, the percentage of cells with 3 spots increased (3rd column in Table 2). In these 3-spot cells the distributions of individual spots in the rifampicin-treated cells are similar to those in the control (results not shown). The nucleoid organization in the 3-spot cells is also very similar to that in the untreated cells, the L-O-R configuration being found in 74% of the rifampicin cells (data not shown) vs. 77% for the control cells (Table 1A, column I). Due to continued replication, the percentage of cells with 4 spots (only the origin replicated) and 5 spots decreased dramatically causing an increase in the percentage of 6-spot cells (6th column in Table 2).

To better compare the patterns in cells with replicated arms (6 spots), Table 2 shows the same numbering of columns VI–X as used in Table 1B. The percentages of cells in Table 2 showing either one (column VI or IX) or two origin spots (column VII) lying outside of the Left- and Right-arm loci have decreased in comparison to the control cells, whereas the percentage of cells showing the LOR LOR pattern (column VIII) or other patterns with the origin spot between the Left and Right arm (column X) have increased. The percentages in columns IX and X reflect the same trend as in the control. Apparently, the patterns in columns VIII and X can be considered to be a final stage of segregation, while the patterns in column VI, VII, and IX reflect a class of cells that is still in the process of segregation.

In view of (i) the significantly decreased percentage of cells with 4 or 5 spots, (ii) the absence of cells with adjacent LL, OO, RR spots, and (iii) the decrease in percentage of cells showing the origin lying outside of the other loci (columns VI and VII in Table 2), we conclude that chromosome movements in non-growing cells occur in a similar way as in growing cells, placing the two arms in different halves of the nucleoid with the origin in between.

### Analysis of segregation distances between spot pairs (LL, RR) and loci on different arms (LR)

The schematic cell images included in Tables 1, 2 suggest a seemingly ordered, lengthwise distribution of duplicated loci. However, in many cells the two groups of duplicated L-, O-, and R-spots are irregularly positioned along the length axis (see Figure 1B). To get a better insight in the positioning and movement of the loci, we determined the distances over which duplicated spots, i.e., spot pairs replicated from the same chromosome arm (L-L and R-R distances), have migrated to opposite cell halves. Depending on the spot pattern and thus the Left/Right arm configuration, the distances between spot pairs will differ. But how will the segregation distance between spot pairs differ in dependence of the distance of the loci from the origin? Table 3 shows that for the LRLR pattern these distances remain more or less constant in the first 5 constructs. For the RLLR pattern the R-R distances tend to increase, while the L-L distances decrease with their distance from the origin. For the LRRL pattern the opposite behavior is observed (see also Figure S3). For all arm configurations the distances in the last construct (FH4060) are smaller. A possible explanation is that these terminus-proximal loci remain tethered together by the terminus region (see Espéli et al., 2012).

**Table 3:** **Average distances between spot pairs (LL, OO and RR) in 6-spot cells for the three ordering patterns of Left (L) and Right (R) chromosome arms in the different constructs^a^**.

**Strains**	**Mean length 6-spot cells (μm)**	**Number 6-spot cells**	**LRLR**				**RLLR**				**LRRL**			
			**%**	**Mean distance spot pairs (μm)**			**%**	**Mean distance spot pairs (μm)**			**%**	**Mean distance spot pairs (μm)**		
				**L-L**	**O-O**	**R-R**		**L-L**	**O-O**	**R-R**		**L-L**	**O-O**	**R-R**
4056^b^ 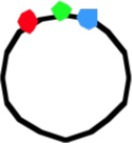	3.80 ± 0.78	625	65	1.39 ± 0.54	1.43 ± 0.55	1.33 ± 0.53	14	1.12 ± 0.52	1.43 ± 0.57	1.67 ± 0.60	21	1.83 ± 0.6	1.54 ± 0.64	1.18 ± 0.51
4057^b^ 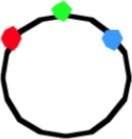	4.04 ± 0.87	326	72	1.49 ± 0.51	1.63 ± 0.54	1.49 ± 0.50	17	1.11 ± 0.44	1.76 ± 0.65	1.91 ± 0.61	12	2.10 ± 0.7	1.63 ± 0.68	1.06 ± 0.51
4035^c^ 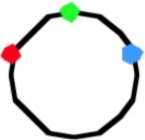	3.02 ± 0.49	420	57	1.28 ± 0.44	1.49 ± 0.43	1.29 ± 0.47	17	0.96 ± 0.36	1.57 ± 0.46	1.73 ± 0.53	26	1.68 ± 0.5	1.50 ± 0.42	0.91 ± 0.34
4058^b^ 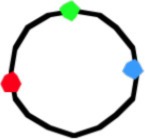	3.89 ± 0.71	243	68	1.51 ± 0.57	1.77 ± 0.57	1.46 ± 0.55	12	0.85 ± 0.44	1.86 ± 0.65	2.21 ± 0.79	20	2.26 ± 0.5	1.91 ± 0.53	0.98 ± 0.34
4059^b^ 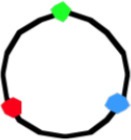	3.89 ± 0.75	123	65	1.47 ± 0.50	1.53 ± 0.47	1.44 ± 0.50	24	0.81 ± 0.25	1.55 ± 0.55	1.97 ± 0.54	11	1.92 ± 0.5	1.69 ± 0.52	0.68 ± 0.32
4060^b^ 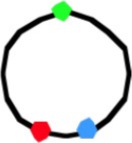	3.85 ± 0.61	131	70	1.01 ± 0.46	1.79 ± 0.51	1.10 ± 0.40	16	0.66 ± 0.29	1.68 ± 0.57	1.41 ± 0.60	15	1.37 ± 0.5	1.83 ± 0.49	0.67 ± 0.39
4035+rifampicin^d^ 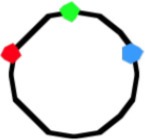	2.73 ± 0.48	1036	52	1.07 ± 0.36	1.12 ± 0.38	1.07 ± 0.35	30	0.64 ± 0.28	1.09 ± 0.37	1.45 ± 0.42	18	1.51 ± 0.4	1.08 ± 0.41	0.72 ± 0.27

a *Spot patterns were qualified using the ObjectJ qualification mode, (cf. Table 1B)*.

b *Cells grown at 32°C; doubling time Td = ~150 min*.

c *Cells grown at 28°C (Td = 180 min) showing smaller cell lengths (see Materials and Methods)*.

d *The data of three populations treated with rifampicin have been averaged (cf. Table 2)*.

As to be expected, in cells with the RLLR or LRRL configuration, the mean distance between spot pairs on the outer arms is significantly larger than on the inner arms (see Table 3). For the LRLR ordering the mean segregation distance between the origins (O-O distance) is slightly larger (1.57 μm) than between the loci on either the Right arm (R-R distance) or the Left arm (L-L distance), which are similar for the first 5 constructs (Table 3).

For the rifampicin-treated cells (last row in Table 3) it can be seen that all segregation distances are about 0.3 μm smaller than in the growing cells, and cell length is also 0.3 μm smaller. The percentage of cells showing the LRLR ordering pattern has decreased from 57% in the control cells of FH4035, to an average of 52% in the FH4035 cultures treated with rifampicin (Table 3). This could indicate a more random segregation with respect to whether either the leading or the lagging strand moves faster (Wang et al., 2005) in growth-inhibited cells.

We conclude that in the majority of cells showing the LRLR configuration, spot pairs segregate over equal distances independent of their distance from the origin (Figure S3). This is in accordance to the movements of chromosome arms as previously described by Nielsen et al. (2006a,b) and Wang et al. (2006). Also in rifampicin-treated cells with the LRLR configuration different spot pairs segregate during run-off DNA synthesis over equal but slightly shorter distances. In contrast, the L-R distances show an increase as a function of the time of replication, except for the latest replicated loci (68 min; Figure 2 and Tables S2, S3). In the Discussion below we will consider these results in the light of two proposed segregation models, the doughnut- and the sausage-model.

## Discussion

### Segregation in non-growing cells

The main conclusion from the present study is that segregation of chromosome arms continues in non-growing cells during run-off DNA replication. This result falsifies a previous proposal that the process of transertion drives DNA segregation (Woldringh, 2002) and supports the hypothesis that segregation is merely driven by the process of *de novo* DNA synthesis and accumulation. Ideas of a replication-driven segregation have been proposed previously by Grossman (Lemon and Grossman, 2001), Hansen (Nielsen et al., 2006a,b), Sherratt (Wang et al., 2006), Austin (Nielsen et al., 2007; Youngren et al., 2014), and Wiggins (Wiggins et al., 2010). A direct link between DNA replication and chromosome organization has been demonstrated and emphasized by the group of Sherratt (Liu et al., 2010), who also presented evidence that transertion played no role in *E. coli* chromosome segregation (Wang and Sherratt, 2010).

### Doughnut and sausage models for segregation

Two different models have been suggested for explaining observations on the position and movement of fluorescent DNA loci in *E. coli* cells, the doughnut model (Niki et al., 2000) and the sausage model (Wang et al., 2006; Liu et al., 2010).

**Figure 4:**
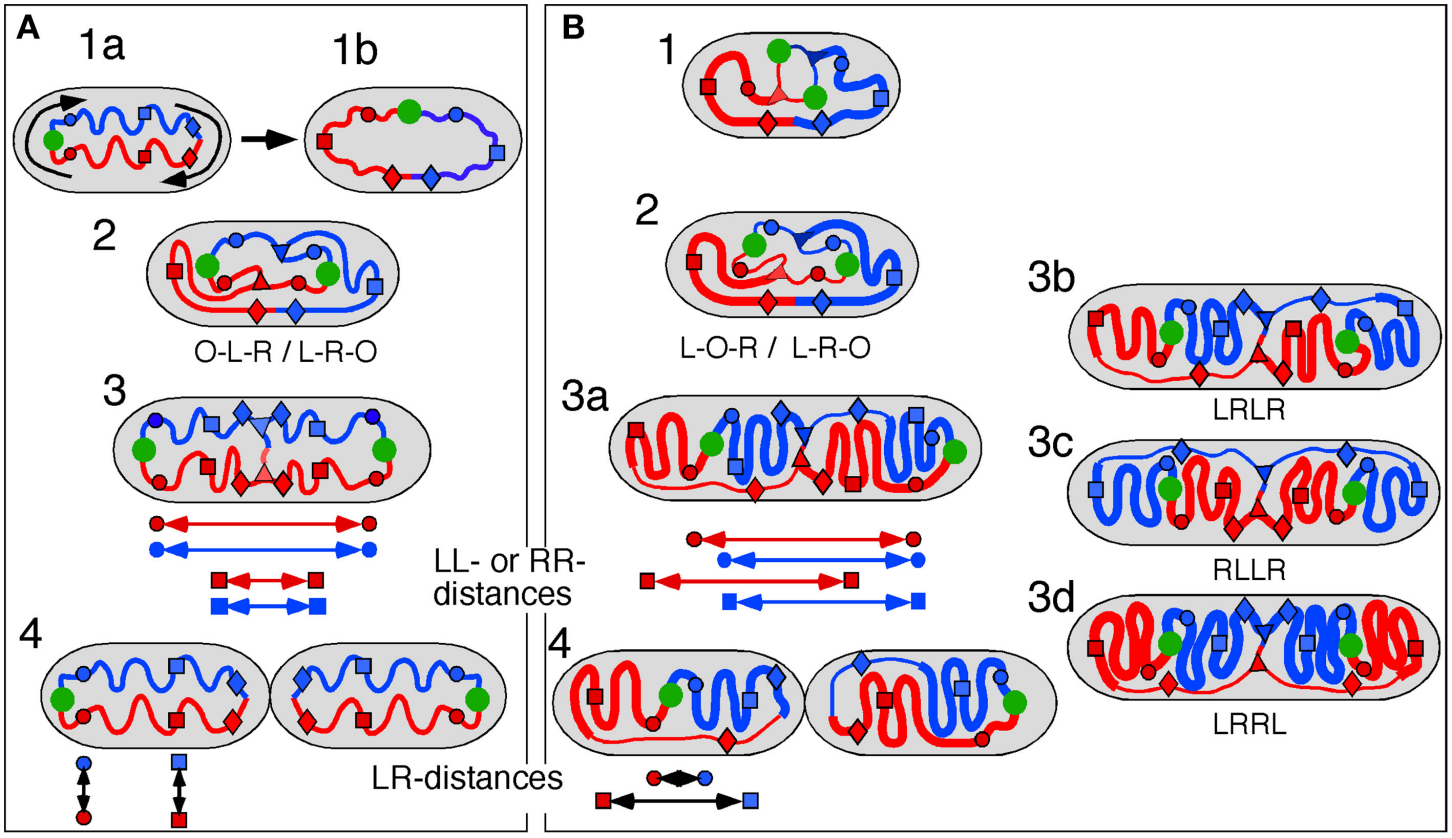
**Schematic representation of doughnut and sausage models**. Green circle, origin; red and blue circles, origin-proximal loci; red and blue squares, origin-distal loci; red and blue diamonds, terminus-proximal loci; red and blue triangles, replisomes; double arrows indicate distances between loci. **(A)** Doughnut model. The drawings are based on the assumption of parallel, symmetric deposition of the daughter strands leading to a configuration with both origins at the end of the nucleoid (OLR/LRO; panels 3, 4). The circular nucleoid (panel 1a) has to be re-arranged to bring the origin and terminus to the cell center before replication initiates (panel 1b). The model illustrates distances between spot pairs (LL-distances; panel 3) decreasing with their distance from the origin, whereas LR-distances (panel 4) remain constant for origin-proximal and origin-distal loci. **(B)** Sausage model. Replicating chromosome leading to both LOR and LRO configurations (panel 2). The drawings are based on the assumption of an alternating, asymmetric deposition of the daughter strands, which requires stretched regions of replicated DNA to feed the newly developing nucleoids (thin lines). The model illustrates equal distances between spot pairs (LL-distances; panels 3a,b) and an increase in LR-distance for origin-distal loci (panel 4). Cells with the arm configuration LRLR, show LL- and RR-distances that are the same (cf. panels 3a,b); cells with the LRRL configuration show that the LL-distance increases and the RR-distance decreases with distance from the origin (panel 3c); cells with the RLLR configuration, show the LL-distance to decrease and the RR-distance to increase with distance from the origin (panel 3d).

In the doughnut model newly replicated DNA is deposited in parallel by the replisomes resulting in a separation of the arms in the short axis of the cell (Figure 4A, panels 2, 3). This mode of segregation is reminiscent of the situation in *Caulobacter* (Toro and Shapiro, 2010), but has also been observed in fast-growing *E. coli* cells (Youngren et al., 2014).

As depicted for the doughnut model (Figure 4A), the replicated origins move to and remain positioned at the polar ends of the developing nucleoid until division (Figure 4A, panel 4). In rapidly growing cells this polar position of the origins and the parallel orientation of the chromosome arms in the short axis is maintained during the next round of replication. In these cells replication occurs at their quarter positions (see Figure 7 in Youngren et al., 2014), giving rise to a branched doughnut structure with partial overlap of chromosome arms of different replication cycles. However, in slow-growing cells, replication initiates in the center of the cell (Reyes-Lamothe et al., 2008). The doughnut model therefore requires a reorientation of the origin by a “sliding movement” that places the two chromosome arms in the two halves of the nucleoid along the cell length axis with the origin in the cell center (Niki et al., 2000; see Figure 4A, panels 1a,b). The developing nucleoid arms have the earliest replicated loci (colored circles in Figure 4) moved further apart than later replicated loci (colored squares in Figure 4). This causes a decrease in the distance between replicated loci pairs (LL and RR) with their distance from the origin (Figure 4A, panel 3). In contrast, the distances between loci on the parallel arms (L-R-distances) remain constant (Figure 4A, panel 4).

In the sausage model, depicted in Figure 4B, the replicated origins remain in the center of the nucleoid (Figure 4B, panel 2), although a polar positioning can also be observed (Table 1A, column II; see also Figure 4B, panel 3a). To obtain the alternating orientation of chromosome arms in the long axis, newly replicated DNA of each arm is layered to both inner and outer edges of the newly developing nucleoids. This implies that newly replicated DNA of one arm passes the origin and the other arm, possibly by means of a thread-like structure (Figure 4B, panels 2, 3b). In this way, both the earliest and the later replicated loci show similar distances between loci pairs (LL and RR), independent of their distance from the origin (Figure 4B, arrows in panel 3a). In contrast, the distances between loci on either arm (LR-distance) increase with their distance from the origin (Figure 4B, arrows in panel 4).

### Comments on the doughnut model

As discussed above, the doughnut model requires a rearrangement of the ring-shaped chromosome before initiation of replication (Niki et al., 2000). A somewhat different reorganization has been proposed by Fisher et al. (2013). In their model, initiation of DNA replication first starts at the polar origin region before one of the daughter strands switches place with unreplicated, parental DNA, placing the terminus in the central part of the nucleoid (see Figure 5B in Fisher et al., 2013). Our data on the 4 spot cells argue against this proposal as we observed very few cells with the OORL configuration even for the most origin proximal RL pair (Table 1A).

The high percentage of cells showing the origin outside of the loci on one or two arms (columns II, IIIB and V in Table 1A and VI and VII in Table 1B) would support the doughnut model (Figure 4A). However, an origin lying outside the other loci (OLR-pattern) can still be envisaged to occur from asymmetric, alternating deposition of chromosome arms (see Figure 4B, right cell half in panels 2–4). In the 3-spot cells of Figure S2B showing the RLO-pattern, the long axis distributions of L- and R-loci are not fully overlapping as to be expected for the doughnut model (Figure 4A, panel 1a).

### Comments on the sausage model

In the sausage model (Liu et al., 2010; Wiggins et al., 2010) the alternating deposition of replicated DNA to the inner and outer edges of the newly developing nucleoids requires that at least one of the replicated chromosome arms passes the other arm (and the origin). This remote deposition could occur through a thread-like structure (thin lines as depicted in Figure 4B). We do not know whether such “feeding threads” exist or how much DNA they contain. They may be revealed by a faster (Brownian) movement of DNA segments through these narrow threads. In the case of a LRLR arm configuration, each replisome forms feeding threads of unequal length resulting in an alternating arm pattern (Figure 4B, panel 3b). Presently, indications for the existence of such threads are still lacking.

## Concluding remarks

The results in Table 3 (see also Figure S3) show that the distances measured between loci pairs (LL and RR) for the different arm configurations stay either constant (LRLR) or show an increase or decrease (LRRL or RLLR) as predicted by the sausage model (Figure 4B, panel 3a). Likewise, the distances between loci on the two arms (LR-distances) in the non-replicating (3 spot) cells showing an increase as a function of their distance from the origin (Figure 3), conform clearly to the sausage model (Figure 4B, panel 4) rather than to the doughnut model (Figure 4A, panel 1b). In addition, it should be noted that distributions of spots in the short axis of the present cells did not show the bi-modality as expected for the doughnut model (Figure 4A) and as observed in the wide cells of Youngren et al. (2014) (results not shown).

The large variation in the ordering patterns of loci in all constructs suggests that the position and orientation of the nucleoid in the cell is not fixed, so sometimes the origin can lie outside the L and R loci, with a thin thread of DNA extending to the origin close to one pole (Figure 4B, panel 3a). This variation is in agreement with the images of nucleoid shape and dynamics obtained by Fisher et al. (2013). Such a flexibility of nucleoid organization and loci positioning is necessary when considering the shape and size changes cells undergo during nutritional shift-up.

At some point during the transition to multifork replication and wider cells, the linear, sausage-like nucleoid will obtain a ring-shaped doughnut structure. Future theoretical work on ring- and linear-polymers will help to understand how, during nutritional shift-up, these two structures move from one state into one another. At present, it only seems clear that in both structures the duplicated origins are separated spontaneously along the cell's long axis by *de novo* DNA synthesis and accumulation and thermodynamic demixing of the newly replicated strands (Youngren et al., 2014).

Segregation can be considered to occur in two stages, (i) the separation of daughter strands or replicated loci as considered here in growing and non-growing cells and (ii) the division of the nucleoid. Because under conditions of inhibited growth nucleoid divisions do not seem to occur (Figure 3) it remains possible that an active mechanism like transertion or cell constriction functions in the second stage, the division of the nucleoid in growing cells (see also discussion in Woldringh and Nanninga, 2006).

### Conflict of interest statement

The authors declare that the research was conducted in the absence of any commercial or financial relationships that could be construed as a potential conflict of interest.

## References

[B1] Cunha S. Woldringh C. L. Odijk T. H. (2001). Polymer-mediated compaction and internal dynamics of isolated *Escherichia coli* nucleoids. J. Struct. Biol. 136, 53–66. 10.1006/jsbi.2001.4420 11858707

[B2] Cunha S. Woldringh C. L. Odijk T. H. (2005). Restricted diffusion of DNA segments within the isolated *Escherichia coli* nucleoid. J. Struct. Biol. 150, 226–232. 10.1016/j.jsb.2005.02.004 15866745

[B3] Espéli O. Borne R. Dupaigne P. Thiel A. Gigant E. Mercier R.. (2012). A MatP-divisome interaction coordinates chromosome segregation with cell division in *E. coli*. EMBO J. 31, 3198–3211. 10.1038/emboj.2012.128 22580828 PMC3400007

[B4] Fisher J. K. Bourniquel A. Witz G. Weiner B. Prentiss M. Kleckner N. (2013). Four-dimensional imaging of *E. coli* nucleoid organization and dynamics in living cells. Cell 153, 882–895. 10.1016/j.cell.2013.04.006 23623305 PMC3670778

[B5] Fritsche M. Li S. Heermann D. W. Wiggins P. A. (2011). A model for *Escherichia coli* chromosome packaging supports transcription factor-induced DNA domain formation. Nucleic Acids Res. 40, 972–9801-9. 10.1093/nar/gkr779 21976727 PMC3273793

[B6] Huls P. G. Vischer N. O. E. Woldringh C. L. (1999). Delayed nucleoid segregation in *Escherichia coli*. Mol. Microbiol. 33, 959–970. 10.1046/j.1365-2958.1999.01535.x 10476030

[B7] Jun S. Wright A. (2010). Entropy as the driver of chromosome segregation. Nat. Rev. Microbiol. 8, 600–607. 10.1038/nrmicro2391 20634810 PMC3148256

[B8] Lark K. G. (1972). Evidence for the direct involvement of RNA in the initiation of DNA replication in *Escherichia coli* 15T-. J. Mol. Biol. 64, 47–60. 10.1016/0022-2836(72)90320-8 4552485

[B9] Le Chat L. Espéli O. (2012). Let's get “Fisical” with bacterial nucleoid. Mol. Microbiol. 86, 1285–1290. 10.1111/mmi.12073 23078263

[B10] Lemon K. P. Grossman A. D. (2001). The extrusion-capture model for chromosome partitioning in bacteria. Genes Dev. 15, 2031–2041. 10.1101/gad.913301 11511534

[B11] Liu X. Wang X. Reyes-Lamothe R. Sherratt D. (2010). Replication-directed sister chromosome alignment in *Escherichia coli*. Mol. Microbiol. 75, 1090–1097. 10.1111/j.1365-2958.2009.06791.x 20487299 PMC2859247

[B12] Luijsterburg M. S. Noom M. C. Wuite G. J. Dame R. T. (2006). The architectural role of nucleoid-associated proteins in the organization of the bacterial chromatin: a molecular perspective. J. Struct. Biol. 156, 262–272. 10.1016/j.jsb.2006.05.006 16879983

[B13] Maaløe O. Hanawalt P. C. (1961). Thymine deficiency and the normal DNA replication cycle. I. J. Mol. Biol. 3, 144–155. 10.1016/S0022-2836(61)80041-7 13764647

[B14] Mason D. J. Powelson D. M. (1956). Nuclear division as observed in live bacteria by a new technique. J. Bacteriol. 71, 474–479. 13319263 10.1128/jb.71.4.474-479.1956 PMC357828

[B15] Michelsen O. Teixeira de Mattos M. J. Jensen P. R. Hansen F. G. (2003). Precise determinations of C and D periods by flow cytometry in *Escherichia coli* K-12 and B/r. Microbiology 149, 1001–1010. 10.1099/mic.0.26058-0 12686642

[B16] Nielsen H. J. Li Y. Youngren B. Hansen F. G. Austin S. J. (2006a). Progressive segregation of the *Escherichia coli* chromosome. Mol. Microbiol. 61, 383–393. 10.1111/j.1365-2958.2006.05245.x 16771843

[B17] Nielsen H. J. Ottesen J. R. Youngren B. Austin S. J. Hansen F. G. (2006b). The *Escherichia coli* chromosome is organized with the left and right chromosome arms in separate cell halves. Mol. Microbiol. 62, 331–338. 10.1111/j.1365-2958.2006.05346.x 17020576

[B18] Nielsen H. J. Youngren B. Hansen F. G. Austin S. (2007). Dynamics of *Escherichia coli* chromosome segregation during multifork replication. J. Bacteriol. 189, 8660–8666. 10.1128/JB.01212-07 17905986 PMC2168957

[B19] Niki H. Yamaichi Y. Hiraga S. (2000). Dynamic organization of chromosomal DNA in *Escherichia coli*. Genes Dev. 14, 212–223. 10.1101/gad.14.2.212 10652275 PMC316355

[B20] Odijk T. (1998). Osmotic compaction of supercoiled DNA into a bacterial nucleoid. Biophys. Chem. 73, 23–29. 10.1016/S0301-4622(98)00115-X 9697298

[B21] Pelletier J. Halvorsen K. Ha B.-Y. Paparcone R. Sandler S. J. Woldringh C. L.. (2012). Physical manipulation of the *Escherichia coli* chromosome reveals its soft nature. Proc. Natl. Acad. Sci. U.S.A. 109, E2649–E2656. 10.1073/pnas.1208689109 22984156 PMC3479577

[B22] Reyes-Lamothe R. Possoz C. Danilova O. Sherratt D. J. (2008). Independent positioning and action of *Escherichia coli* replisomes in live cells. Cell 133, 90–102. 10.1016/j.cell.2008.01.044 18394992 PMC2288635

[B23] Skarstad K. Boye E. Steen H. B. (1986). Timing of initiation of chromosome replication in individual *Escherichia coli* cells. EMBO J. 5, 1711–1717. 3527695 10.1002/j.1460-2075.1986.tb04415.x PMC1166998

[B24] Toro E. Shapiro L. (2010). Bacterial chromosome organization and segregation. Cold Spring Harb. Perspect. Biol. 2:a000349. 10.1101/cshperspect.a000349 20182613 PMC2828278

[B25] Wang X. Liu X. Possoz C. Sherratt D. J. (2006). The two *Escherichia coli* chromosome arms locate to separate cell halves. Genes Dev. 20, 1727–1731. 10.1101/gad.388406 16818605 PMC1522069

[B26] Wang X. Possoz C. Sherratt D. J. (2005). Dancing around the divisome: asymmetric chromosome segregation in *Escherichia coli*. Genes Dev. 19, 2367–2377. 10.1101/gad.345305 16204186 PMC1240045

[B27] Wang X. Reyes-Lamothe R. Sherratt D. J. (2008). Modulation of *Escherichia coli* sister chromosome cohesion by topoisomerase IV. Genes Dev. 22, 2426–2433. 10.1101/gad.487508 18765793 PMC2532930

[B28] Wang X. Sherratt D. J. (2010). Independent segregation of the two arms of the *Escherichia coli ori* region requires neither RNA synthesis nor MreB dynamics. J. Bacteriol. 192, 6143–6153. 10.1128/JB.00861-10 20889756 PMC2981198

[B29] Wiggins P. A. Cheveralls K. C. Martin J. S. Lintner R. Kondev J. (2010). Strong intranucleoid interactions organize the *Escherichia coli* chromosome into a nucleoid filament. Proc. Natl. Acad. Sci. U.S.A. 107, 4991–4995. 10.1073/pnas.0912062107 20194778 PMC2841910

[B30] Woldringh C. L. (2002). The role of co-transcriptional translation and protein translocation (transertion) in bacterial chromosome segregation. Mol. Microbiol. 45, 17–29. 10.1046/j.1365-2958.2002.02993.x 12100545

[B31] Woldringh C. L. Nanninga N. (2006). Structural and physical aspects of bacterial chromosome segregation. J. Struct. Biol. 156, 273–283. 10.1016/j.jsb.2006.04.013 16828313

[B32] Yamaichi Y. Niki H. (2004). *migS*, a cis-acting site that affects bipolar positioning of *oriC* on the *Escherichia coli* chromosome. EMBO J. 23, 221–233. 10.1038/sj.emboj.7600028 14685268 PMC1271666

[B33] Yazdi N. H. Guet C. C. Johnson R. C. Marko J. F. (2012). Variation of the folding and dynamics of the *Escherichia coli* chromosome with growth conditions. Mol. Microbiol. 86, 1318–1333. 10.1111/mmi.12071 23078205 PMC3524407

[B34] Youngren B. Nielsen H. J. Jun S. Austin S. (2014). The multifork *Escherichia coli* chromosome is a self-duplicating and self-segregating thermodynamic ring polymer. Genes Dev. 28, 71–84. 10.1101/gad.231050.113 24395248 PMC3894414

[B35] Zechiedrich E. L. Khodursky A. B. Bachellier S. Schneider R. Chen D. Lilley D. M. J.. (2000). Roles of topoisomerases in maintaining steady-state DNA supercoiling in *Escherichia coli*. Biol. Chem. 275, 8103–8113. 10.1074/jbc.275.11.8103 10713132

