# Supplementary material for: Segregation of chromosome arms in growing and non-growing Escherichia coli cells
Source: Front Microbiol. 2015 May 12;6:448. doi: 10.3389/fmicb.2015.00448 (PMC4428220; doi:10.3389/fmicb.2015.00448)
Supplement: Supplementary file 1 [file Table1.DOCX]

| *parS* sequence | Template | Location on chromosome (a) | Oligo-nucleotides | Oligonucleotide sequence (b) |
| --- | --- | --- | --- | --- |
| pMT1 | pFH3615 | *asnA/asnC*  3.925/84.6/0 | 28.20  28.21 | **tccagtcgttggcggtcatgattgtcatgctcattaacaatgaccaaaccAGGAAGCGGAATTCCGGACC**  **tgtgttgaggttgcatggacggttaagccgagaatacggtagtaagtgagCTCGAGATGCAGAAGACGCA** |
| P1 | pFH3614 | *yrhB/ggt*  3.583/77.2/7.3 | 26.27  26.28 | **aggctaccttcggcttgccctgacaaaatagccctcttcccacgaagaggAGGAAGCGGAATTCCGGACC**  **ccgacccgcgctcggtggatgatttaacggcggggtactaaggttagcggCTCGAGATGCAGAAGACGCA** |
|  |  | In *yhaV*  3.275/70.6/14.0 | 26.17  26.18 | **ggcgctatatgctcatccctgttttcaggaaacctacgacgctttagttgAGGAAGCGGAATTCCGGACC**  **gatgatggattgaccgtgatatgctcctcaatcactttatggactaccgcCTCGAGATGCAGAAGACGCA** |
|  |  | *yfiO/raiA*  2.735/58.9/25.6 | 24.10  24.11 | **caatacataacagaaacctgaaacacaaaacggcagcccttgagctgccgAGGAAGCGGAATTCCGGACC**  **tgatgagatcgatagcgactaaatcgcttcagtttcacaactgacagaatCTCGAGATGCAGAAGACGCA** |
|  |  | *yfaD/yfaU*  2.356/50.8/33.8 | 24.06  24.07 | **cggctgcggcaggaaggacatcaaattggctggcaggaaggtaaattagaAGGAAGCGGAATTCCGGACC**  **tcgctgccagatcggcttcgcttagctgggtggccgcgagcacctggtcaCTCGAGATGCAGAAGACGCA** |
|  |  | In *yoaI*  1.872/40.3/44.2 | 27.09  27.10 | **cggtccgttcgataagaagaacggacaaaaccagtacaacagcaatggcAGGAAGCGGAATTCCGGACC**  **catgctatgaacgatcaaatgtttgtcgagacactgattatcacgtcatcCTCGAGATGCAGAAGACGCA** |
| P7 | pFH3616 | *yafS/rnhA*  0.235//5.1/20.5 | 16.25  16.26 | **aggcggttggagccacccggcaatgtcgtaaaccacaggcttaaAGGAAGCGGAATTCCGGACC**  **tgaatcccacactggaagatacaggctaccaagttgaagtttaaCTCGAGATGCAGAAGACGCA** |
|  |  | *malE/malK*  4.245/91.5/6.9 | 16.19  16.20 | **cggcaacctctttccatcctccttgcccctacgccccaccAGGAAGCGGAATTCCGGACC**  **cattaccgccaattctgtaacagagatcacacaaagcgacCTCGAGATGCAGAAGACGCA** |
|  |  | *nanC/fimB*  4.538/97.8/13.2 | 17.19  17.20 | **atatgtttcctggtttgtggcttgtaactggtcacttctgAGGAAGCGGAATTCCGGACC**  **ctgaaaacaccaacatcaacaagcctctccagatcgacttCTCGAGATGCAGAAGACGCA** |
|  |  | *tesB/ybaY* 0.474/10.2/25.6 | 17.25  17.26 | **gctactggtccgatgggtgcaatggtctgaattacgggctAGGAAGCGGAATTCCGGACC**  **caacaagtgtggcacacatcacgcatttctgcctgtaattCTCGAGATGCAGAAGACGCA** |
|  |  | *ompX/ybiP* 0.850/18.3/33.8 | 16.21  16.22 | **tgccggtgttggttaccgcttctaatcactttggtgatatAGGAAGCGGAATTCCGGACC**  **catctcggaaccgatattttcgacccgaaaccttaaaaacCTCGAGATGCAGAAGACGCA** |
|  |  | *hns/tdk* 1.292/27.8/43.3 | 17.15  17.16 | **acagtcgcaataagagcatggacttagtattgcactatctAGGAAGCGGAATTCCGGACC**  **aacctgttgcgcaagtaatagccctctgttgacctccaggCTCGAGATGCAGAAGACGCA** |
