# Supplementary material for: Segregation of chromosome arms in growing and non-growing Escherichia coli cells
Source: Front Microbiol. 2015 May 12;6:448. doi: 10.3389/fmicb.2015.00448 (PMC4428220; doi:10.3389/fmicb.2015.00448)
Supplement: Supplementary file 2 [file Table1.PDF]

Table S-1. Oligonucleotides used to construct the strains shown in figure 1.

parS sequence	Template	Location on chromosome (a)	Oligo-nucleotides	Oligonucleotide sequence (b)
pMT1	pFH3615	*asnA/asnC*	28.20	**tccagtcgttggcggtcatgattgtcatgctcattaacaatgaccaaaccAGGAAGCGGAATTCCGGACC**
		3.925/84.6/0	28.21	**tgtgttgaggttgcatggacggttaagccgagaatacggtagtaagtgagCTCGAGATGCAGAAGACGCA**
P1	pFH3614	*yrhB/ggt*	26.27	**aggctaccttcggcttgccctgacaaaatagccctcttcccacgaagaggAGGAAGCGGAATTCCGGACC**
		3.583/77.2/7.3	26.28	**ccgacccgcgctcggtggatgatttaacggcggggtactaaggttagcggCTCGAGATGCAGAAGACGCA**
		In *yhaV*	26.17	**ggcgctatatgctcatccctgttttcaggaaacctacgacgctttagttgAGGAAGCGGAATTCCGGACC**
		3.275/70.6/14.0	26.18	**gatgatggattgaccgtgatatgctcctcaatcactttatggactaccgcCTCGAGATGCAGAAGACGCA**
		yfiO/raiA	24.10	**caatacataacagaaacctgaaacacaaaacggcagcccttgagctgccgAGGAAGCGGAATTCCGGACC**
		2.735/58.9/25.6	24.11	**tgatgagatcgatagcgactaaatcgcttcagtttcacaactgacagaatCTCGAGATGCAGAAGACGCA**
		yfaD/yfaU	24.06	**cggctgcggcaggaaggacatcaaattggctggcaggaaggtaaattagaAGGAAGCGGAATTCCGGACC**
		2.356/50.8/33.8	24.07	**tcgctgccagatcggcttcgcttagctgggtggccgcgagcacctggtcaCTCGAGATGCAGAAGACGCA**
		In *yoaI*	27.09	**cggtccgttcgataagaagaacggacaaaaccagtacaacagcaatggcAGGAAGCGGAATTCCGGACC**
		1.872/40.3/44.2	27.10	**catgctatgaacgatcaaatgtttgtcgagacactgattatcacgtcatcCTCGAGATGCAGAAGACGCA**
P7	pFH3616	*yafS/rnhA*	16.25	**aggcggttggagccacccggcaatgtcgtaaaccacaggcttaaAGGAAGCGGAATTCCGGACC**
		0.235//5.1/20.5	16.26	**tgaatcccacactggaagatacaggctaccaagttgaagtttaaCTCGAGATGCAGAAGACGCA**
		malE/malK	16.19	**cggcaacctctttccatcctccttgcccctacgccccaccAGGAAGCGGAATTCCGGACC**
		4.245/91.5/6.9	16.20	**cattaccgccaattctgtaacagagatcacacaaagcgacCTCGAGATGCAGAAGACGCA**
		nanC/fimB	17.19	**atatgtttcctggtttgtggcttgtaactggtcacttctgAGGAAGCGGAATTCCGGACC**
		4.538/97.8/13.2	17.20	**ctgaaaacaccaacatcaacaagcctctccagatcgacttCTCGAGATGCAGAAGACGCA**
		tesB/ybaY 0.474/10.2/25.6	17.25	**gctactggtccgatgggtgcaatggtctgaattacgggctAGGAAGCGGAATTCCGGACC**
			17.26	**caacaagtgtggcacacatcacgcatttctgcctgtaattCTCGAGATGCAGAAGACGCA**
		ompX/ybiP 0.850/18.3/33.8	16.21	**tgccggtgttggttaccgcttctaatcactttggtgatatAGGAAGCGGAATTCCGGACC**
			16.22	**catctcggaaccgatattttcgacccgaaaccttaaaaacCTCGAGATGCAGAAGACGCA**
		hns/tdk 1.292/27.8/43.3	17.15	**acagtcgcaataagagcatggacttagtattgcactatctAGGAAGCGGAATTCCGGACC**
			17.16	**aacctgttgcgcaagtaatagccctctgttgacctccaggCTCGAGATGCAGAAGACGCA**

(a) Location on the chromosome is given as mB / min on *E. coli* map / min from *oriC*. (b) The sequences are presented with small and capital letters. Small letters is the oligonucleotide sequence homologous to the sequence at the position on the chromosome where the *parS* sequence is to be inserted. Capital letters is the sequence homologous to the template plasmid providing the *parS* sequence and the *cat* gene.
