# Supplementary material for: Segregation of chromosome arms in growing and non-growing Escherichia coli cells
Source: Front Microbiol. 2015 May 12;6:448. doi: 10.3389/fmicb.2015.00448 (PMC4428220; doi:10.3389/fmicb.2015.00448)

Table S2. Average relative distances between L- and R-loci for qualified 3 spot cells. Compare with columns I and II in Table 1A and with Fig. 2.

| Strains  time of replication (min) and  chromosome position | Qualification: LOR | | Qualification: OLR / ORL | |
| --- | --- | --- | --- | --- |
|  | Mean length  (cell number) | relative LR-dist.  (±SD) | Mean length  (cell number) | relative LR-dist.  (±SD) |
| FH4056  (11')  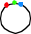 | 2.61  (325) | 0.21±0.25 | 2.56  (202) | 0.13 ±0.08 |
| FH4057  (21')  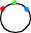 | 2.75  (360) | 0.32±0.11 | 2.62  (127) | 0.19±0.10 |
| FH4035  (30')  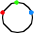 | 2.07  (295) | 0.35±0.11 | 2.09  (89) | 0.23±0.09 |
| FH4058  (39')  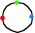 | 2.61  (480) | 0.49±0.15 | 2.55  (137) | 0.24±0.13 |
| FH4059  (51')  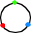 | 2.75  (208) | 0.50±0.16 | 2.75  (240) | 0.25±0.13 |
| FH4060  (67')  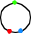 | 2.48  (153) | 0.40±0.16 | 2.37  (152) | 0.23±0.11 |


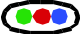

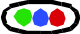

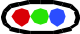

Supplement: Supplementary file 3 [file Table2.DOCX]
