# Supplementary material for: Segregation of chromosome arms in growing and non-growing Escherichia coli cells
Source: Front Microbiol. 2015 May 12;6:448. doi: 10.3389/fmicb.2015.00448 (PMC4428220; doi:10.3389/fmicb.2015.00448)
Supplement: Supplementary file 4 [file Table2.PDF]

Table S2. Average relative distances between L- and R-loci for qualified 3 spot cells. Compare with columns I and II in Table 1A and with Fig. 2.

Strain time of replication (min)[(a)]	Qualification: LOR 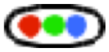		Qualification: OLR / ORL 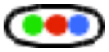 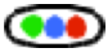	
	mean length (cell number)	relative LR-dist.(±SD)	Mean length (cell number)	relative LR-dist.(±SD)
FH4056 (11') 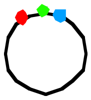	2.61 (325)	0.21±0.25	2.56 (202)	0.13 ±0.08
FH4057 (21') 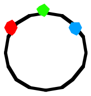	2.75 (360)	0.32±0.11	2.62 (127)	0.19±0.10
FH4035 (30') 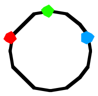	2.07 (295)	0.35±0.11	2.09 (89)	0.23±0.09
FH4058 (39') 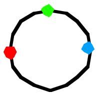	2.61 (480)	0.49±0.15	2.55 (137)	0.24±0.13
FH4059 (51') 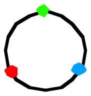	2.75 (208)	0.50±0.16	2.75 (240)	0.25±0.13
FH4060 (67') 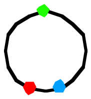	2.48 (153)	0.40±0.16	2.37 (152)	0.23±0.11

(a) For "time of replication" see legend to **Figure 1A**.
