# Supplementary material for: Segregation of chromosome arms in growing and non-growing Escherichia coli cells
Source: Front Microbiol. 2015 May 12;6:448. doi: 10.3389/fmicb.2015.00448 (PMC4428220; doi:10.3389/fmicb.2015.00448)
Supplement: Supplementary file 5 [file Table3.PDF]

Table S3. Average relative distances between O spot pairs and R and L spots in 4-spot cells for the four different ordering patterns. Spot distances were calculated relative to average cell length of the cells having the indicated pattern.

Strains	Mean cell length (µm)	No. 4-spot cells	Mean spot distances in all cells		% cells different patterns				Mean spot distances							
					LOOR	OORL/ OOLR	OLOR/ OROL	OLRO	LOOR 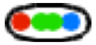		OORL/ OOLR 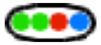		OLOR/ OROL 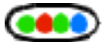		OLRO 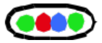	
			O-O	L-R					O-O	L-R	O-O	L-R	O-O	L-R	O-O	L-R
FH4056 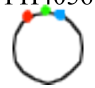	3.08	63	0.16	0.16	35	6	40	19	0.11	0.22	0.13	0.07	0.18	0.15	0.21	0.10
FH4057 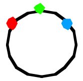	3.20	137	0.22	0.24	33	10	42	15	0.15	0.31	0,17	0.15	0.25	0.24	0.30	0.15
FH4035 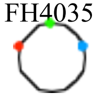	2.31	130	0.28	0.32	36	2	36	26	0.19	0.43	0.20	0.16	0.31	0.31	0.37	0.17
FH4058 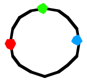	3.09	190	0.31	0.53	44	8	35	13	0.22	0.62	0.23	0.40	0.38	0.51	0.49	0.37
FH4059 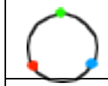	3.29	56	0.33	0.42	41	7	32	20	0.24	0.59	0.30	0.25	0.35	0.41	0.48	0.13
FH4060 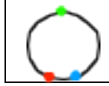	2.96	412	0.38	0.28	14	11	43	32	0.25	0.48	0.31	0.16	0.38	0.33	0.49	0.17
