# Supplementary material for: Segregation of chromosome arms in growing and non-growing Escherichia coli cells
Source: Front Microbiol. 2015 May 12;6:448. doi: 10.3389/fmicb.2015.00448 (PMC4428220; doi:10.3389/fmicb.2015.00448)
Supplement: Supplementary file 6 [file DataSheet1.DOCX]

***Supplementary Material***

**Segregation of chromosome arms in growing and non-growing *Escherichia coli* cells**

Conrad L. Woldringh ^*1^, Flemming G. Hansen^2^, Norbert O.E. Vischer^1^, Tove Atlung^3^

^1^ Bacterial Cell Biology, Swammerdam Institute for Life Sciences, Faculty of Science, University of Amsterdam, Kruislaan 316, 1098 SM Amsterdam, The Netherlands

^2^ Department of Systems Biology, Technical University of Denmark, Matematiktorvet, Building 301, DK-2800 Kgs. Lyngby, Denmark

^3^ Department of Science, Systems and Models, Roskilde University, Universitetsvej 1, P.O. Box 260, DK-4000 Roskilde

*** Correspondence** C.L. Woldringh, Swammerdam Institute for Life Sciences, University of Amsterdam, Science Park 904, 1098 XH Amsterdam, the Netherlands

c.woldringh@gmail.com

1. Strain construction

2.1. Supplemantary Tables: Table S-1, S-2, S-3

2.2. Supplementary Figures: Figure S1, S2, S3

3. References

**1.1. Strain construction**

Plasmids carrying three different *parS* sequences were constructed as follows: Restriction enzyme fragments carrying the respective *parS* sequences were recovered on *BamHI-BlpI* fragments from plasmids pTK533 (P1 *parS*; obtained from Kenn Gerdes), pALA1840 (pMT1 *parS*; Youngren et al. 2000), and pALA1993 (P7 *parS*; obtained from Stuart Austin), respectively, and inserted into plasmid pHJN2 next to a cat gene flanked by *loxP* sites (Nielsen et al., 2006b), resulting in plasmids pFH3214 (P1 *parS*), pFH3228 (pMT1 *parS*), and pFH3521 (P7 *parS*).. To avoid problems in future P1 transductions we replaced the cat gene between *loxP* sites with a *cat* gene between FRT sites. The FRT-*cat*-FRT fragment was generated on a PCR-fragment using the oligonucleotides 26.12 and 26.13 containing the FRT sequence and with plasmid pHJN2 as template for the cat gene. Plasmids pFH3614 (P1 *parS*), pFH3615 (pMT1 *parS*), and pFH3616 (P7 *parS*) were constructed by restricting the *loxP parS* *loxP* containing plasmids mentioned above and the FRT-cat-FRT PCR fragment with XmaI restriction enzyme, followed by ligation, and checking with restriction enzyme analysis.

The *Escherichia coli* strain FH2927 is a Δ*lacIZYA* derivative of MG1655 and was used to construct strains with three different *parS* sequences on the chromosome. First, the pMT1 *parS* sequence and a *cat* gene located between two perfect FRT sites was recovered on a PCR fragment and, using the primers shown in Table S1, recombineered in very close to the origin of replication between the *asnA* and *asnC* genes selecting for chloramphenicol resistance. The *cat* gene was removed by transforming with the temperature sensitive plasmid pCP20 (Cherepanov and Wackernagel, 1995) carrying the *flp* recombinase gene. Secondly, six strains were constructed by recombineering the P7 *parS* and *cat* sequences into this strain at different positions on the right replichore; again selecting chloramphenicol resistance and removing the *cat* gene with plasmid pCP20. Thirdly, six strains were constructed by recombineering the P1 *parS* and *cat* genes into positions on the left replichore of strain FH2927 followed by P1 transduction of P1 *parS* and *cat* sequences into the strains which already carried the pMT1 *parS* and the P7 *parS* sequences in the chromosome selecting for chloramphenicol resistance or in one case for kanamycin resistance (See Table S1 for oligonucleotides used for strain construction). Finally plasmid pFH4034 was transformed into these strains which could then be used to follow three differently colored spots on the chromosome. The construction of plasmid pFH4035 is described in **Figure S1.**
