# Supplementary material for: Segregation of chromosome arms in growing and non-growing Escherichia coli cells
Source: Front Microbiol. 2015 May 12;6:448. doi: 10.3389/fmicb.2015.00448 (PMC4428220; doi:10.3389/fmicb.2015.00448)
Supplement: Supplementary file 7 [file DataSheet2.DOCX]

**3. References**

Cherepanov, P.P. and W. Wackernagel (1995) Gene disruption in *Escherichia coli:* TcR and KmR cassettes with the option of Flp-catalyzed excision of the antibiotic-resistance determinant. Gene 158: 9-14.

Nielsen, H.J., Li, Y., Youngren, B., Hansen, F.G. and Austin, S.J. (2006a) Progressive segregation of the *Escherichia coli* chromosome. Mol. Microbiol. 61, 383-393.

[Youngren, B](http://www.ncbi.nlm.nih.gov/pubmed?term=Youngren%20B%5BAuthor%5D&cauthor=true&cauthor_uid=10869068)., [Radnedge](http://www.ncbi.nlm.nih.gov/pubmed?term=Radnedge%20L%5BAuthor%5D&cauthor=true&cauthor_uid=10869068), L., [Hu](http://www.ncbi.nlm.nih.gov/pubmed?term=Hu%20P%5BAuthor%5D&cauthor=true&cauthor_uid=10869068), P., [Garcia](http://www.ncbi.nlm.nih.gov/pubmed?term=Garcia%20E%5BAuthor%5D&cauthor=true&cauthor_uid=10869068), E., [Austin](http://www.ncbi.nlm.nih.gov/pubmed?term=Austin%20S%5BAuthor%5D&cauthor=true&cauthor_uid=10869068), S., 2000. A plasmid partition system of the P1-P7par family from the pMT1 virulence plasmid of Yersinia pestis. [J Bacteriol.](http://www.ncbi.nlm.nih.gov/pubmed/10869068) 182, 3924-3928.
