# Supplementary material for: Segregation of chromosome arms in growing and non-growing Escherichia coli cells
Source: Front Microbiol. 2015 May 12;6:448. doi: 10.3389/fmicb.2015.00448 (PMC4428220; doi:10.3389/fmicb.2015.00448)
Supplement: Supplementary file 8 [file DataSheet3.DOCX]

**2.2. Supplementary Figures**

**Supplementary Figure S1.** Plasmid pFH4034 was constructed as follows: First, the P1 parB sequence of plasmid pFH2973 (Nielsen et al. 2006) was replaced by a P7 parB sequence originating from pALA1571 (Youngren et al., 2000); secondly, the P1 parB-mCherry gene was recovered on a PCR fragment from plasmid pALA2487 (Therese Brendler, unpublished) and inserted between the CFP-P7 parB and YGFP-pMT1 parB genes.
