# Supplementary material for: Segregation of chromosome arms in growing and non-growing Escherichia coli cells
Source: Front Microbiol. 2015 May 12;6:448. doi: 10.3389/fmicb.2015.00448 (PMC4428220; doi:10.3389/fmicb.2015.00448)
Supplement: Supplementary file 9 [file DataSheet4.DOCX]

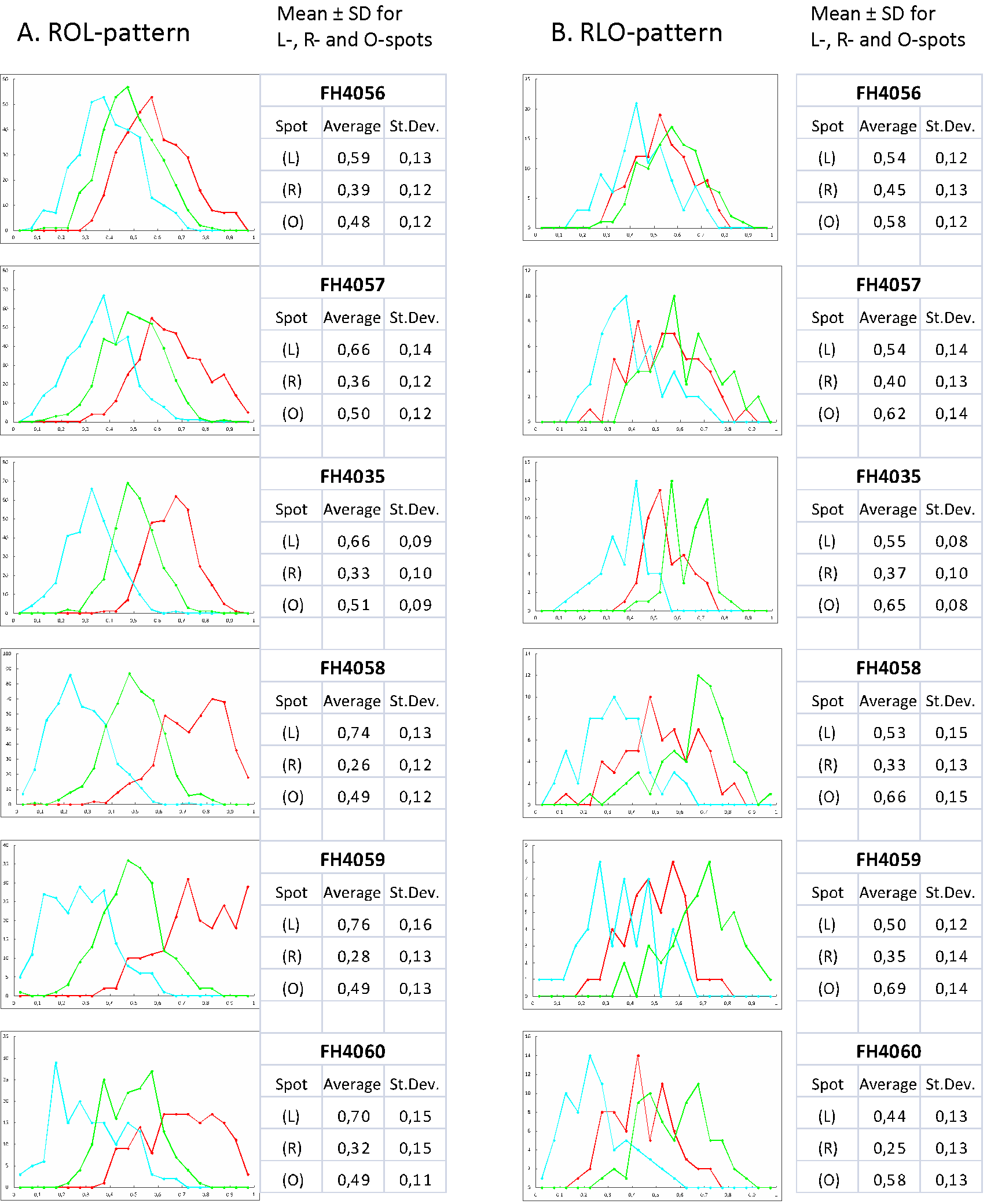


**Supplementary Figure S2.** Distributions of spot positions in the various constructs along the normalized cell length axis for 3-spot cells. The cyan-lines represent spots on the right chromosome arm (R) and the red lines spots on the left chromosome arm (L). The green lines represent the origin (O). Statistics are given to the right**.** **(A)** Cells showing the ROL (LOR) pattern have been ordered so that cyan spots (R) lie to the left of the origin spots (O), which lie to the left of the red spots (L). **(B)** Cells showing the RLO pattern have been ordered so that cyan spots (R) lie to the left of the red spots (L), which lie to the left of the origin spots (O).

**
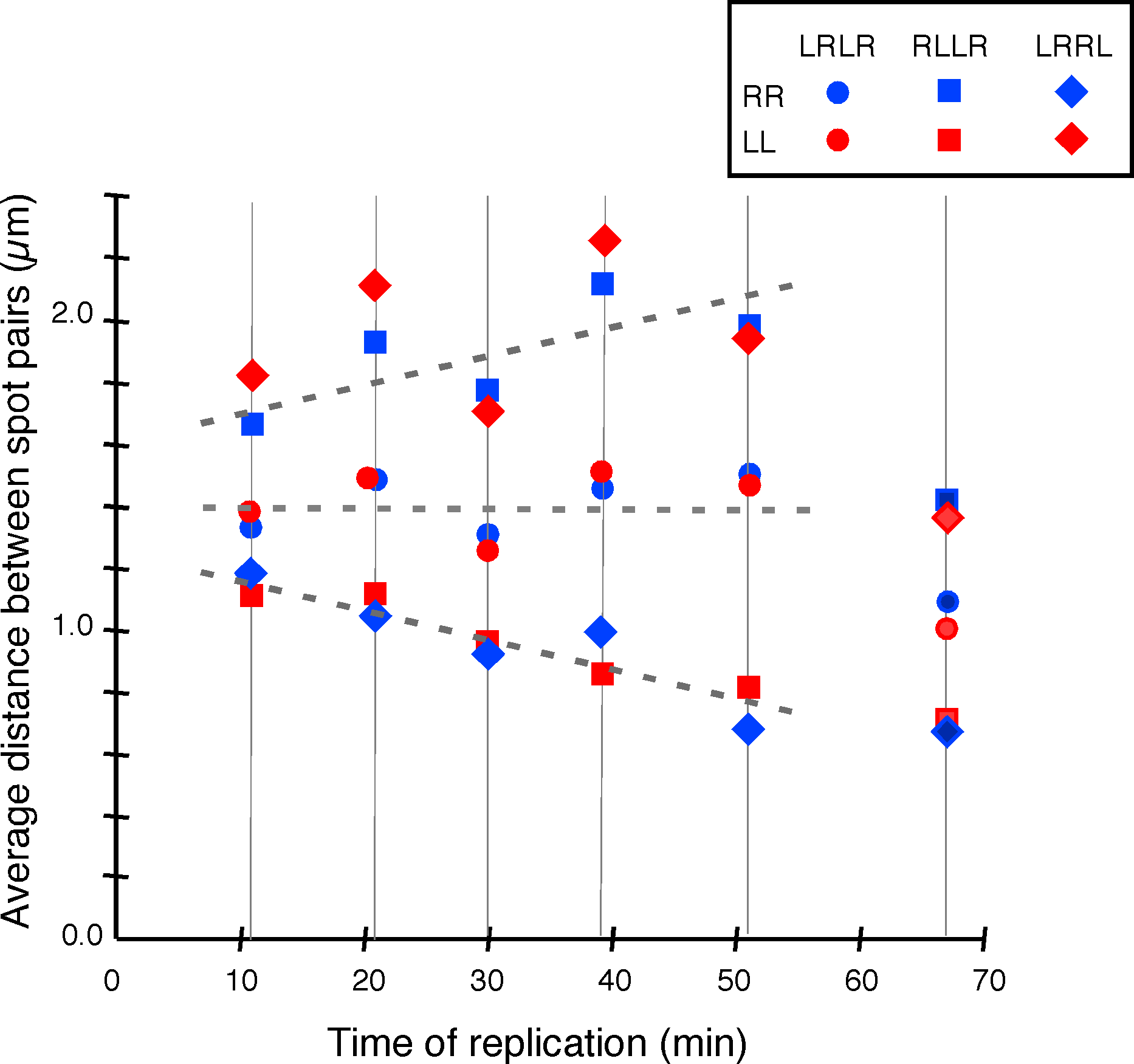
**

**Supplementary Figure S3.** Average distances between spot pairs (LL, RR) in 6-spot cells for the three configurations of chromosome arms as a function of time of replication. Data are taken from Table 3. For the LRLR pattern (blue and red circles) the average distances remain relatively constant at ~1.41 µm, decreasing only in the last construct (cf. **Figure 4B**, panel 3b). For the RLLR pattern, the RR distances (blue squares) increase with the distance from the origin, while the LL distances (red squares) decrease (see **Figure 4B**, panel 3c). The LRRL pattern (red and blue diamonds) shows the reverse behavior (see **Figure 4B**, panel 3d).

**3. References**

Cherepanov, P.P. and W. Wackernagel (1995) Gene disruption in *Escherichia coli:* TcR and KmR cassettes with the option of Flp-catalyzed excision of the antibiotic-resistance determinant. Gene 158: 9-14.

Nielsen, H.J., Li, Y., Youngren, B., Hansen, F.G. and Austin, S.J. (2006a) Progressive segregation of the *Escherichia coli* chromosome. Mol. Microbiol. 61, 383-393.

[Youngren, B](http://www.ncbi.nlm.nih.gov/pubmed?term=Youngren%20B%5BAuthor%5D&cauthor=true&cauthor_uid=10869068)., [Radnedge](http://www.ncbi.nlm.nih.gov/pubmed?term=Radnedge%20L%5BAuthor%5D&cauthor=true&cauthor_uid=10869068), L., [Hu](http://www.ncbi.nlm.nih.gov/pubmed?term=Hu%20P%5BAuthor%5D&cauthor=true&cauthor_uid=10869068), P., [Garcia](http://www.ncbi.nlm.nih.gov/pubmed?term=Garcia%20E%5BAuthor%5D&cauthor=true&cauthor_uid=10869068), E., [Austin](http://www.ncbi.nlm.nih.gov/pubmed?term=Austin%20S%5BAuthor%5D&cauthor=true&cauthor_uid=10869068), S., 2000. A plasmid partition system of the P1-P7par family from the pMT1 virulence plasmid of Yersinia pestis. [J Bacteriol.](http://www.ncbi.nlm.nih.gov/pubmed/10869068) 182, 3924-3928.
